# Retinal Neurodegeneration in Different Risk Phenotypes of Diabetic Retinal Disease

**DOI:** 10.3389/fnins.2021.800004

**Published:** 2021-12-21

**Authors:** Maria H. Madeira, Inês P. Marques, Sónia Ferreira, Diana Tavares, Torcato Santos, Ana Rita Santos, João Figueira, Conceição Lobo, José Cunha-Vaz

**Affiliations:** ^1^AIBILI, Association for Innovation and Biomedical Research on Light and Image, Coimbra, Portugal; ^2^Faculty of Medicine, Coimbra Institute for Clinical and Biomedical Research, University of Coimbra, Coimbra, Portugal; ^3^Center for Innovative Biomedicine and Biotechnology, University of Coimbra, Coimbra, Portugal; ^4^Department of Orthoptics, School of Health, Polytechnic of Porto, Porto, Portugal; ^5^Department of Ophthalmology, Centro Hospitalar e Universitário de Coimbra, Coimbra, Portugal

**Keywords:** diabetes, retinopathy, neurodegeneration, progression, personalized medicine, risk phenotypes

## Abstract

Diabetic retinopathy (DR) has been considered a microvascular disease, but it has become evident that neurodegeneration also plays a key role in this complex pathology. Indeed, this complexity is reflected in its progression which occurs at different rates in different type 2 diabetic (T2D) individuals. Based on this concept, our group has identified three DR progression phenotypes that might reflect the interindividual differences: phenotype A, characterized by low microaneurysm turnover (MAT <6), phenotype B, low MAT (<6) and increased central retinal thickness (CRT); and phenotype C, with high MAT (≥6). In this study, we evaluated the progression of DR neurodegeneration, considering ganglion cell+inner plexiform layers (GCL+IPL) thinning, in 170 T2D individuals followed for a period of 5 years, to explore associations with disease progression or risk phenotypes. Ophthalmological examinations were performed at baseline, first 6 months, and annually. GCL+IPL average thickness was evaluated by optical coherence tomography (OCT). Microaneurysm turnover (MAT) was evaluated using the RetMarkerDR. ETDRS level and severity progression were assessed in seven-field color fundus photography. In the overall population there was a significant loss in GCL+IPL (−0.147 μm/year), independently of glycated hemoglobin, age, sex, and duration of diabetes. Interestingly, this progressive thinning in GCL + IPL reached higher values in phenotypes B and C (−0.249 and −0.238 μm/year, respectively), whereas phenotype A remained relatively stable. The presence of neurodegeneration in all phenotypes suggests that it is the retinal vascular response to the early neurodegenerative changes that determines the course of the retinopathy in each individual. Therefore, classification of different DR phenotypes appears to offer relevant clarification of DR disease progression and an opportunity for improved management of each T2D individual with DR, thus playing a valuable role for the implementation of personalized medicine in DR.

## Introduction

Diabetic retinopathy (DR) is a leading cause of vision loss and preventable blindness worldwide and the most common microvascular complication of diabetes (Ting et al., 2016), causing significant disability that threatens independence with a major impact on life quality (Narayan et al., 2006). Its progression occurs at different rates in different individuals, with some developing major vision-threatening complications that lead to vision loss, diabetic macular edema (DME), and proliferative diabetic retinopathy (PDR) (Cunha-Vaz et al., 2014).

Indeed, these inter-individual alterations are of major relevance when studying DR, its biomarkers, and possible therapeutic strategies. Diabetic retinopathy has been considered a microvascular disease, characterized by microaneurysms and dot blot hemorrhages, with these alterations forming the base for its current grade classification, diagnosis, and therapeutic strategies. Based on this concept, retinal microvascular alterations identified by the turnover rate of microaneurysms (MAs) and retinal structural alterations, namely central retinal thickness (CRT), allowed the identification of three phenotypes of non-proliferative DR (NPDR) (Nunes et al., 2013), which have been shown to be associated with the development of vision-threatening complications (Marques et al., 2020b) and disease severity progression (Marques et al., 2021b). Individuals with low microaneurysm turnover (MAT; <6) and normal CRT are classified as phenotype A, whereas individuals with low MAT (<6) and increased CRT are classified as phenotype B, and individuals with MAT >6, independently of the CRT, are classified as phenotype C (Nunes et al., 2013; Ribeiro et al., 2018).

Importantly, DR is now understood as a complex disease, in which, besides microvascular alterations, neurodegeneration also appears as a relevant disease pathway (Simó et al., 2018; Marques et al., 2019, 2020a,b). In diabetic individuals, the use of optical coherence tomography (OCT) facilitated the identification of these neurodegenerative changes. Thinning of the ganglion cell layer + inner plexiform layer (GCL+IPL) and retinal nerve fiber layer (RNFL) assessed in serial OCT examination and reflecting retinal neurodegenerative changes have been shown to occur in early stages of DR and in a progressive manner (Chhablani et al., 2015; Kim et al., 2019; Aschauer et al., 2020; Lim et al., 2020). In fact, it has been suggested that the DR neurodegenerative processes result from diabetes-induced neuro-glial activation, which leads to reduced neuronal function and apoptosis, proceeding to microvascular impairment in certain individuals (Lieth et al., 2000; Sohn et al., 2016).

However, most of the studies resulted from cross-sectional analyses or involved short-term follow-up periods, which might not be sufficient to perform a complete evaluation of these changes. Of note, recent findings from our group appeared to indicated that DR microvascular and neurodegenerative events could occur independently (Marques et al., 2019, 2020a). However, the association between these disease components, their correlation with long-term progression, and the relation with specific DR risk phenotypes remains an open question. The study here presented was designed to further characterize the retinal neurodegenerative events in DR, by assessing the retinal layer thickness by OCT, focusing on GCL+IPL thinning (considered as neurodegeneration), over a 5-year follow-up period in type 2 diabetic (T2D) individuals in the early stages of disease. These changes were then correlated with demographic and clinical features of the disease in order to explore possible associations with disease progression or risk phenotypes.

## Materials and Methods

This prospective longitudinal observational cohort study, the PROGRESS study (ClinicalTrials.gov identifier: NCT03010397) (Marques et al., 2020b), was designed to conduct a 5-year follow-up of individuals with T2D (T2D) and mild NPDR (level 20 or 35 on the Early Treatment Diabetic Retinopathy Study [ETDRS] severity scale) at an ophthalmological level. The tenets of the Declaration of Helsinki were followed, and approval from the AIBILI’s Ethics Committee for Health with the number CEC/007/16 was obtained. Each participant signed written informed consent agreeing to participate in the study.

Individuals were included according to specified inclusion and exclusion criteria, and were followed up at 6 months and then annually for a 5-year period or until they developed vision-threatening complications as DME or PDR (Marques et al., 2020b). Exclusion criteria included (1) glycated hemoglobin A1c (HbA_1c_) level >10% (85.8 mmol/mol); (2) any previous laser treatment or intravitreal injections; (3) presence of vitreomacular disease, age-related macular degeneration, or glaucoma; (4) high ametropia (spherical equivalent greater than −6 and +2 diopters); and (5) any other systemic disease that could affect the eye, with special attention for uncontrolled systemic hypertension and history of ischemic heart disease. Eyes with baseline CRT identifying center involved macular edema (CIME) defined as a retinal thickness (RT) ≥290 μm in women and ≥305 μm in men (Friedman et al., 2015) were also excluded.

For each participant, the baseline registry of demographic data was performed, including gender, age, duration of diabetes, physical and biometric measures (body weight and height), blood pressure evaluation, and blood analysis with determination of glycated HbA_1c_ and lipid profile, as previously reported (Marques et al., 2020b). The remaining study visits were performed annually or at the last visit before treatment (in eyes that developed one of the endpoints).

At all study visits individuals underwent a complete ophthalmological examination of the study eye including best-correct visual acuity (BCVA, using the ETDRS protocol and Precision Vision charts at 4 m), slit-lamp examination, intraocular pressure (IOP) measurement, digital seven-field color fundus photography (CFP), and OCT.

A total of 212 individuals with T2D were included in the study, and one eye per person selected at baseline according to the inclusion/exclusion criteria. When both eyes fulfilled the criteria, the one showing the more advanced ETDRS severity level was chosen as the study eye. Of those, 145 eyes completed the 5-year follow-up and 27 developed one or more of the study endpoints (Marques et al., 2020b). A total of 170 individuals were considered for data analysis, as 2 individuals were excluded due to missing data on some variables of the initial visit. A population of 58 healthy control individuals was used as reference for baseline demographic variables and structural OCT evaluation.

### Color Fundus Photography and Early Treatment Diabetic Retinopathy Study Classification

Color fundus photographs (CFP) were performed according to the ETDRS protocol and under mydriasis. The seven-field photographs were obtained at 30/35°, using a Topcon TRC 50DX mydriatic camera (Topcon Medical Systems, Tokyo, Japan). The DR severity score was determined at baseline and every annual visit by two independent graders in a context of an experienced reading center (Coimbra Ophthalmology Reading Center – CORC, Coimbra, Portugal) using a modified Airlie House classification scheme according to the ETDRS Protocol (Soares et al., 2017; Early Treatment Diabetic Retinopathy Study Research Group, 2020). The observed agreement between the two graders was 97%. All disagreement cases were resolved by mutual agreement (Figueira et al., 2018).

Step changes in the ETDRS retinopathy severity scale were used to describe worsening or improvement of the retinopathy (Klein et al., 2001; Early Treatment Diabetic Retinopathy Study Research Group, 2020), and were determined as the difference between levels of ETDRS at baseline and at the 5-year follow-up and classified as improvement or worsening according to the reduction or increase of the retinopathy severity level, as previously described (Marques et al., 2021b).

### Microaneurysm Quantification

Automated microaneurysm (MA) quantification was obtained based on the analyses of 45/50° two-field images, using the RetmarkerDR (Retmarker SA, Coimbra, Portugal), as previously described (Oliveira et al., 2011; Cunha-Vaz et al., 2012; Marques et al., 2021b). Briefly, this automated computer-aided diagnostic system identifies MA and red-dot-like vascular lesions in the macula (all referred to as MAs), and for each eye it computes the number of MAs in each visit and the number of MAs that appear and/or disappear from one visit to the other. This approach allows the calculation of MAT, as the sum of the MA formation and disappearance rates, determined at the 6-month visit.

### Optical Coherence Tomography and Retinal Layers Segmentation

Optical coherence tomography (OCT) was performed using the Cirrus HD-OCT 5000 (Carl Zeiss Meditec, Inc., Dublin, CA, United States), applying the acquisition protocol Macular Cube 512 × 128, which consists of 128 B-scans with 512 A-scans each, and was used to assess the subjects’ CRT.

Retinal layer segmentation was gathered with Zeiss Cirrus standard reports, for central retinal thickness (CRT).

Segmentation of retinal layers to assess the average thickness value of the RNFL, GCL+IPL, inner nuclear layer (INL), outer plexiform layer (OPL), outer nuclear layer and inner segments (ONL+IS), outer segments (OS), and retinal pigmented epithelium (RPE), at the inner ring, was performed using the segmentation software implemented by AIBILI (Marques et al., 2020a,b). Automated analysis results were reviewed by a masked grader.

### Phenotype Definition

The three different DR phenotypes for NPDR, previously described by our group (Nunes et al., 2013; Cunha-Vaz et al., 2014), were identified in the study eyes at the 6-month study visit. This classification is based on MAT and CRT according to the following rules: phenotype A: MAT <6 and normal CRT values (central subfield RT <260 μm for women and <275 μm in men, i.e., normal mean ± 1 SD); phenotype B: MAT <6 and increased CRT values (CRT ≥260 μm for women and ≥275 μm in men); phenotype C: MAT ≥6, with or without increased CRT. Central retinal thickness reference values presented are the reference for Zeiss Cirrus SD-OCT (Friedman et al., 2015; Lobo et al., 2018).

### Best Corrected Visual Acuity Evaluation

Best corrected visual acuity was evaluated and recorded as letters read at 4 m on ETDRS charts. Final BCVA letter score was calculated by adding the number of letters read at 4 m plus 30 (or the number of letters read at 1 m). BCVA was evaluated using the Snellen scale and converted into logarithm units of the minimal angle of resolution (logMAR) (Khoshnood et al., 2010). The presence of any visual loss was recorded.

### Endpoint Definition

The outcomes defined in the study were DME and PDR. DME was considered as the presence, in any study visit, of CIME, as defined above, or center involved macular edema (CSME) defined by the ETDRS group as retinal thickening within 500 μm of the center of the fovea or presence of hard exudates (with thickening of the adjacent retina) within 500 μm of the center of the fovea or thickening of at least one disk area located less than one disk diameter from the center of the fovea (Oliveira et al., 2011). PDR was defined by the presence of abnormal neovessels arising to the retina from the optic nerve or elsewhere. For statistical analysis purposes, study participants were divided as endpoint (DME or PDR) or no endpoint.

### Statistical Analysis

A linear mixed model was applied to study longitudinal changes in the GCL+IPL thickness (model 1, using a restricted maximum likelihood approach) since some patients had missing data for some of the visits. Visit (0–5 years) was used as a continuous fixed variable and baseline CRT, age, diabetes duration, HbA_1c_, and gender were inserted as fixed covariates. Participant was used as a random variable (intercept only) (Sohn et al., 2016; Aschauer et al., 2020; Lim et al., 2020; Silk et al., 2020; Van De Kreekeid et al., 2020).

The model was repeated four times using different groups of participants as an additional fixed variable: phenotype (A; B; and C) – model 2; endpoints (yes; no) – model 3; ETDRS level at baseline (10–20; 35) – model 4; and ETDRS level change between baseline and the last visit (improve; equal; and worse) – model 5. The main effect of group and the interaction between group and visit were tested.

To verify the linear mixed models’ assumptions, homoscedasticity and normality of residuals in the models were visually inspected with residuals vs. predicted and Q-Q plots, respectively (Meteyard and Davies, 2020; Silk et al., 2020). The effects of the predicting variables were described with beta coefficients with 95% confidence intervals.

The comparison of baseline characteristics between patients and healthy participants was performed with a *t*-test for normally distributed continuous variables or Mann–Whitney test for continuous variables not following a normal distribution (age and GCL+IPL thickness), and the chi-squared test for categorical variables (gender).

The comparison of baseline characteristics between patients within each group was performed with *t*-test (groups with two categories) or ANOVA (groups with three categories) for normally distributed continuous variables, or Mann–Whitney test (groups with two categories) or Kruskal–Wallis test (groups with three categories) for continuous variables not following a normal distribution (age, diabetes duration, HbA_1c_, MA turnover, BCVA, CRT, and GCL+IPL thickness), and the chi-squared test for categorical variables (gender, phenotype, ETDRS level, and endpoints). *Post-hoc t*-tests or Mann–Whitney tests were performed for groups with more than two categories if statistically significant effects were observed for continuous variables. For categorical variables, *post-hoc* analyses were based on adjusted standardized residuals. Bonferroni correction was applied for multiple comparisons among group categories.

Data normality was assessed with the Shapiro-Wilk test and visually verified with histogram plots. Normally distributed variables were described as mean ± standard deviation and variables not following normal distribution were described as median (interquartile range, IQ). Categorical variables were described as frequency (percentage).

Statistical significance was considered at α = 0.05. Statistical analyses were conducted in Stata (version 16.1; StataCorp LLC, United States).

## Results

Of the 212 T2D individuals initially included in the study, 172 completed the 5-year period of follow-up or achieved one of the endpoints. Forty participants dropped out of the study (Marques et al., 2020b). A total of 170 T2D individuals were considered for data analysis because 2 participants had missing data on some variables required for the analysis. Of these, 26 (15%) developed an endpoint and, therefore, only 144 (84%) achieved the 5-year follow-up.

The individuals included in the study were in the initial stages of DR (NPDR), with 46 (27%) graded as ETDRS level 10–20 and 124 (73%) graded as ETDRS level 35. After phenotype classification, 63 (37%) individuals were classified with phenotype A, 51 (30%) with phenotype B, and 56 (33%) with phenotype C. After the 5-year period of follow-up or endpoint development, 29 (17%) individuals had shown improvement in the ETDRS severity level, 80 (47%) individuals maintained the ETDRS classification, and 61 (36%) showed worsening of their ETDRS level, corresponding to progression of DR severity (Table 1).

**TABLE 1:** Baseline characteristics of the study population and comparison with the healthy controls.

	Diabetic retinopathy (*n* = 170)	Healthy control population (*n* = 58)	Test value	*p*-value
			
	*n* (%) | mean ± standard deviation | median (interquartile range)			
Age (years)	63.0 (10.0)	42.0 (11.0)	^b^*z* = 10.715	<0.001*
**Gender**				
Male	116 (68.2%)	26 (44.8%)	^c^*X*^2^(1) = 10.087	0.001*
Female	54 (31.8%)	32 (55.2%)		
Diabetes duration (years)	14.0 (10.0)	–	–	–
HbA1c (%)	7.3 (1.8)	–	–	–
MA turnover (6 months)	3.7 (6.2)	–	–	–
**Phenotype**				
A	63 (37.1%)	–	–	–
B	51 (30.0%)	–		
C	56 (32.9%)	–		
**ETDRS level**				
10–20	46 (27.1%)	–	–	–
35	124 (72.9%)	–		
**ETDRS change**				
Improved	29 (17.1%)	–	–	–
Maintained	80 (47.1%)	–		
Worsened	61 (35.9%)	–		
**Endpoints**				
No endpoint	144 (84.1%)	–	–	–
Endpoint	26 (15.3%)	–		
Visual acuity (LogMAR)	0.0 (0.1)	–	–	–
CRT thickness (μm)	281.3 ± 21.6	272.0 ± 18.5	^a^*t*(226) = 2.948	0.003*
GCL+IPL thickness (μm)	91.8 (10.6)	94.8 (5.3)	^b^*z* = −2.706	0.007*
RNFL thickness (μm)	24.1 (3.0)	24.9 (3.7)	^b^*z* = −2.133	0.033*
INL thickness (μm)	41.0 (5.7)	39.8 (3.1)	^b^*z* = 1.858	0.063
OPL thickness (μm)	31.1 (6.0)	29.9 (3.6)	^b^*z* = 2.225	0.026*
ONL+IS thickness (μm)	91.3 (3.9)	89.1 (12.1)	^b^*z* = 1.460	0.144
OS thickness (μm)	37.1 (4.4)	37.1 (4.0)	^b^*z* = −0.672	0.502
RPE thickness (μm)	24.5 (3.8)	25.8 (3.7)	^b^*z* = −2.553	0.011*

*Statistical significance values are highlighted in bold, for p < 0.050; ^a^t-test results for continuous parametric variables (represented by mean ± standard deviation); ^b^Mann–Whitney test results for continuous non-parametric variables [represented by median (interquartile range)]; ^c^Chi-square test results for categorical variables (represented by n (%)). HbA1c: Hemoglobin A1c; MA, Microaneurysm; ETDRS, Early Treatment Diabetic Retinopathy; CRT, Central Retinal Thickness; GCL+IPL, Ganglion cell layer and Inner Plexiform Layer; RNFL, Retinal nerve fiber layer; INL, Inner Nuclear Layer; OPL, Outer Plexiform Layer; ONL+IS, Outer Nuclear Layer and Inner Segments; OS, Outer segments; RPE, Retinal Pigmented Epithelium.

Evaluation of retinal structure at baseline (Table 1) showed that the T2D population presented an average CRT of 281.3 ± 21.6 μm, which was statistically significantly higher than the healthy control population, which presented a CRT with 272.0 ± 18.5 μm (*p* = 0.003). No differences were observed in the OS layer between T2D individuals and controls, and there was a significant decrease in the RPE layer thickness in the diabetic population (*p* = 0.011).

Regarding neurodegenerative changes (Table 1), both RNFL and GCL+IPL presented significant thinning in the T2D population when compared with healthy controls. T2D individuals had a median GCL+IPL thickness of 91.8 μm [interquartile range (IQ): 10.6], whereas the healthy control population presented a thickness of 94.8 μm (IQ: 5.3; *p* = 0.007). For RNFL, T2D individuals showed a thickness with a median of 24.1 μm (IQ: 3), with a control population of 24.9 μm (IQ: 3.7; *p* = 0.033).

When looking at the baseline evaluation separating the different DR risk phenotypes, phenotype C presented higher HbA_1c_ levels (*p* < 0.001) than phenotypes A and B, and, as by phenotype definition, higher MAT (*p* < 0.001). Also, phenotype C had more individuals classified with ETDRS level 35 at baseline (*p* < 0.001) (Table 2).

**TABLE 2:** Baseline characteristics of the individuals classified with different DR phenotypes.

	Health control population (*n* = 58)	Phenotype A (*n* = 63)	Phenotype B (*n* = 51)	Phenotype C (*n* = 56)	Test value	*p*-value
			
	*n* (%) | mean ± standard deviation | median (interquartile range)					
Age (years)	42.0 (11.0)	63.4 ± 7.0	64.1 ± 6.9	61.0 ± 7.4	^a^*F*(2, 167) = 2.880	0.059
**Gender**						
Male	26 (44.8%)	43 (68.2%)	36 (70.6%)	37 (66.1%)	^c^*X*^2^(2) = 0.251	0.882
Female	32 (55.2%)	20 (31.7%)	15 (29.4%)	19 (33.9%)		
Diabetes duration (years)	–	11.0 (8.0)	15.0 (11.0)	14.5 (9.0)	^b^*X*^2^(2) = 1.623	0.444
HbA1c (%)	—	7.3 (1.7)	6.5 (1.5)	7.9 (2.2)	^b^*X*^2^(2) = 1.623	<0.001*
MA turnover (6 months)	–	2.0 (3.7)	2.0 (3.9)	12.1 (10.3)	^b^*X*^2^(2) = 112.106	<0.001*
**ETDRS level**						
10–20	–	22 (34.9%)	22 (43.1%)	2 (3.6%)	^c^*X*^2^(1) = 24.305	<0.001*
35	–	41 (65.1%)	29 (56.9%)	54 (96.4%)		
Visual acuity (LogMAR)	–	−0.01 ± 0.07	−0.02 ± 0.08	−0.01 ± 0.08	^a^*F*(2, 167) = 0.240	0.788
CRT thickness (μm)	272.0 ± 18.5	269.3 ± 23.5	300.5 ± 13.8	278.8 ± 30.7	^b^*X*^2^(2) = 64.012	<0.001*
GCL+IPL thickness (μm)	94.8 (5.3)	89.2 ± 7.8	93.9 ± 7.2	91.5 ± 9.1	^a^*F*(2, 167) = 4.790	0.009*
RNFL thickness (μm)	24.9 (3.7)	23.7 (4.6)	24.4 (3.0)	24.2 (2.7)	^c^*X*^2^(2) = 4.459	0.108
INL thickness (μm)	39.8 (3.1)	40.2 (4.9)	42.5 (6.4)	40.6 (4.4)	^c^*X*^2^(2) = 3.595	0.166
OPL thickness (μm)	29.9 (3.6)	29.8 (6.7)	31.3 (5.5)	31.6 (4.5)	^c^*X*^2^(2) = 4.347	0.114
ONL+IS thickness (μm)	89.1 (12.1)	88.8 (13.8)	92.6 (11.8)	90.6 (16.3)	^c^*X*^2^(2) = 6.047	0.049*
OS thickness (μm)	37.1 (4.0)	36.9 (4.2)	37.7 (5.7)	37.2 (3.6)	^c^*X*^2^(2) = 3.813	0.149
RPE thickness (μm)	25.8 (3.7)	24.5 (4.0)	24.2 (4.6)	24.9 (3.7)	^c^*X*^2^(2) = 3.081	0.214

*Statistical significance values between the three phenotypes are highlighted in bold, for p < 0.050; ^a^ANOVA results for continuous parametric variables (represented by mean ± standard deviation); ^b^Kruskal–Wallis test results for continuous non-parametric variables [represented by median (interquartile range)]; ^c^Chi-square test results for categorical variables (represented by n (%)). HbA1c, Hemoglobin A1c; MA, Microaneurysm; CRT, Central Retinal Thickness; GCL+IPL, Ganglion cell layer and Inner Plexiform Layer; RNFL, Retinal nerve fiber layer; INL, Inner Nuclear Layer; OPL, Outer Plexiform Layer; ONL+IS, Outer Nuclear Layer and Inner Segments; OS, Outer segments; RPE, Retinal Pigmented Epithelium.

Regarding retinal structural changes, there was a significant difference between the three phenotypes on CRT thickness (*p* < 0.001), with phenotype B, as by phenotype definition, having the highest value. Significant differences in neurodegenerative response (assessed by GCL+IPL thinning) were also observed between phenotypes (*p* = 0.009), with phenotype A presenting a baseline thickness of 89.2 ± 7.8 μm, lower than phenotype B (93.9 ± 7.2 μm) and phenotype C (91.5 ± 9.1 μm). No significant alterations were observed in the RNFL layer between phenotypes (Table 2).

Using a predictive linear mixed model for GCL+IPL thinning over the 5-year follow-up period, with visit (0–5 years) being the continuous fixed variable and using baseline central retinal thickness, age, diabetes duration, hemoglobin A1c, and gender as fixed covariates, our data show that GCL+IPL thinning had a progression rate of −0.147 μm/year (*p* < 0.001), which was positively associated with CRT (0.126, *p* < 0.001) and negatively associated with age (−0.224, *p* = 0.008). Also, women showed less thinning of GCL+IPL than men (*p* = 0.13) (Table 3).

**TABLE 3:** Predictive model for GCL+IPL thinning over the 5-year follow-up period.

	β-value	β-95% confidence interval		*z*-value	*p*-value
Visit	–0.147	–0.227	–0.067	–3.600	<0.001*
Central retinal thickness (CRT; μm)	0.126	0.068	0.183	4.250	<0.001*
Age (years)	–0.224	–0.390	–0.058	–2.650	0.008*
Gender (female vs. male)	3.381	0.713	6.049	2.480	0.013*
Diabetes duration (years)	–0.058	0.223	0.106	–0.700	0.486
Hemoglobin A1c (%)	0.680	–0.248	1.609	1.440	0.151

Linear mixed model for GCL+IPL thickness in the inner ring (Wald X^2^(10) = 55.680, p < 0.0001*) showing an overall progression rate of −0.147 μm/year, highlighted in gray. *Statistical significance values are highlighted in bold, for p < 0.050; Visit (0–5 years) was used as a continuous fixed variable and baseline central retinal thickness, age, diabetes duration, hemoglobin A1c, and gender were inserted as fixed covariates. Participant was used as a random variable (intercept only). GCL+IPL, Ganglion cell layer and Inner Plexiform Layer.

**FIGURE 1:**
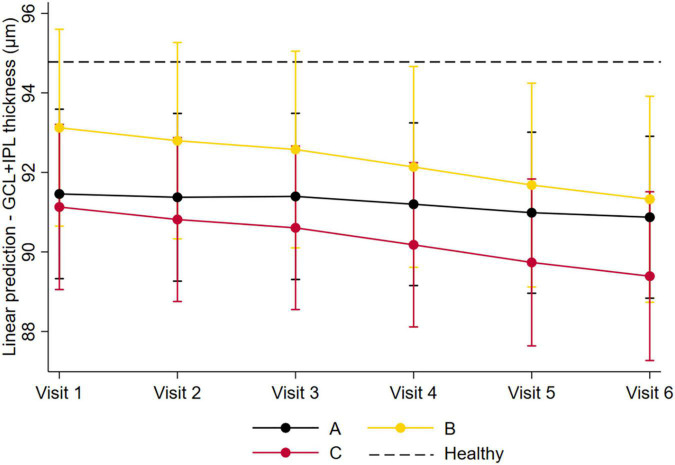
Predicted GCL+IPL thinning over the 5-year follow-up period, between the different DR phenotypes. Graphical representation of the predicted GCL+IPL thinning over the 5-year follow-up period, between the different DR phenotypes, using the linear mixed model. Phenotype A is depicted in black; phenotype B is depicted in yellow; and phenotype C is depicted in red. Dash lines represent baseline GCL+IPL thickness in the healthy control population.

Applying the same predictive linear mixed model for GCL+IPL thinning over the 5-year follow-up period using the DR risk phenotypes (Table 4), patients with phenotype B and C had faster GCL+IPL thinning in comparison with phenotype A (B vs. A: −0.243 μm/year, *p* = 0.014; C vs. A: −0.231 μm/year, *p* = 0.018). Patients with phenotype B did not differ from phenotype C (−0.012 μm/year, *p* = 0.991). As seen in Figure 1, GCL+IPL thickness values in phenotype A remained stable over the 5-year follow-up period, whereas there was marked thinning of this layer in phenotype B and phenotype C. The GCL+IPL thickness of the entire T2D diabetic study population was lower than the baseline GCL+IPL thickness of the healthy control population, indicating that neurodegeneration was present in all diabetic patients and in the different phenotypes.

**TABLE 4:** Predictive model for GCL+IPL thinning (at the inner ring) over the 5-year follow-up period using the DR phenotypes.

	β-value	β-95% confidence interval		*z*-value	*p*-value
Visit	–0.008	–0.132	0.115	–0.130	0.893
Phenotype (B vs. A)^a^	1.665	–1.943	5.274	0.900	0.366
Phenotype (C vs. A)^a^	–0.328	–3.328	2.671	–0.210	0.830
Phenotype (B vs. A) × Visit^b^	–0.243	–0.436	–0.049	–2.460	0.014*
Phenotype (C vs. A) × Visit^b^	–0.231	–0.422	–0.040	–2.370	0.018*
Central retinal thickness (CRT; μm)	0.112	0.038	0.185	2.960	0.003*
Age (years)	–0.240	–0.408	–0.072	–2.790	0.005*
Gender (female vs. male)	3.110	0.314	5.906	2.180	0.029*
Diabetes duration (years)	–0.066	–0.231	0.099	–0.780	0.434
Hemoglobin A1c (%)	0.833	–0.137	1.803	1.680	0.092

Linear mixed model for GCL+IPL thickness in the inner ring using phenotypes (Wald X^2^(10) = 55.680, p < 0.0001*). *Statistical significance values are highlighted in bold, for p < 0.050; and in highlighted in gray are the phenotype B and C progression rates. ^a^Post-hoc contrast between phenotype B and C: β = 1.993 [−1.325; 5.312], z = 1.180, p = 0.239; ^b^Post-hoc contrast between phenotype B and C: β = −0.012 [−0.220; 0.196], z = −0.110, p = 0.911. Visit (0–5 years) was used as a continuous fixed variable, phenotype was used as a categorical fixed variable, and baseline central retinal thickness, age, diabetes duration, hemoglobin A1c, and gender were inserted as fixed covariates. Participant was used as a random variable (intercept only). GCL+IPL, Ganglion cell layer and Inner Plexiform Layer; DR, diabetic retinopathy.

Finally, to confirm the relevance of phenotype classification for retinal neurodegenerative changes in T2D individuals, we explored which variable could be more associated and a better predictor of the GCL+IPL thinning rates, considering phenotype, endpoint development, baseline ETDRS level, and DR severity progression (Table 5). Our data showed that the T2D population, in general, presented a progression rate in GCL+IPL thinning of −0.147 μm/year [95% CI: (−0.227; −0.067)]. When applying the different predicting variables as associated factors, only phenotype B [−0.249 μm/year; 95% CI: (−0.380; −0.118)] and phenotype C [−0.238 mm/year; 95% CI: (−0.399; −0.078)] presented a statistically significant association with increased GCL+IPL thinning rates.

**TABLE 5:** GCL+IPL thinning rates by phenotypes, endpoints, ETDRS levels, and DR severity progression.

	GCL+IPL (μm/year)
All patients	−**0.147 (**−**0.227;**−0.067)*
**Phenotypes**	
A	−0.009 (−0.132; 0.114)
B	−**0.249 (**−**0.380;**−0.118)*^a^
C	−**0.238 (**−**0.399;**−0.078)*^b^
**Endpoints**	
Endpoint	0.026 (−0.342; 0.395)
No endpoint	−0.154 (−0.236; −0.073)
**ETDRS level**	
10–20	−0.136 (−0.262; −0.011)
35	−0.149 (−0.249; −0.050)
ETDRS change*	
Better	−0.152 (−0.303; −0.001)
Equal	−0.193 (−0.303; −0.083)
Worse	−0.084 (−0.240; 0.071)

The effects of the predicting variables are described with beta coefficients with 95% confidence intervals. *Statistical significance values are highlighted in bold for (p < 0.05); ^a^Statistical significance – B > A; ^b^ Statistical significance – C > A; GCL+IPL, Ganglion cell layer and Inner Plexiform Layer; ETDRS, Early Treatment Diabetic Retinopathy; DR, diabetic retinopathy.

## Discussion

Several studies have indicated that neurodegeneration is a disease pathway that is involved in the early pathophysiological events of DR and may precede and therefore be linked to clinically detectable vascular alterations (Sohn et al., 2016; Simó et al., 2018). Indeed, DR has been considered a component of generalized diabetic neuropathy, resulting in the impairment of the so-called retinal “neuro-vascular unit” that causes both neuronal and vascular retinal abnormalities associated with the disease onset and progression (Simó et al., 2018).

In this 5-year longitudinal study we followed up with 170 T2D individuals with minimal DR on fundus examination using OCT image, and we found a significant, progressive loss in GCL + IPL (0.147 μm/year) that was independent of HbA_1C,_ DR grade, progression of DR, or with the development of vision-threatening complications. In agreement with other studies that have reported progressive thinning of GCL+IPL in T2D individuals (Sohn et al., 2016; Kim et al., 2019; Lim et al., 2020), we here confirmed that retinal neurodegenerative changes are a component of DR, and progressive over the course of diabetes.

Interestingly, this 5-year longitudinal study has shown for the first time that these neurodegenerative changes are indeed present in T2D individuals, but with distinct profiles in the different retinopathy phenotypes. Phenotype A showed the presence of neurodegeneration but over the 5-year follow-up period its value remained relatively stable. On the other hand, phenotypes B and C showed a more rapid progression in the thinning of GCL+IPL. It is particularly interesting to note that although the neurodegenerative changes of these two phenotypes showed similar rates of progression, phenotype C was associated with progressive failure in the retinal microvascular response.

This new characterization of the distinct profiles of DR neurogenerative changes identified by the different DR risk phenotypes raises the hypothesis that progression of retinal neurodegeneration in individuals characterized with phenotypes B or C might be the result or consequence of the distinct structural microvascular alterations observed in these two phenotypes, which were not observed in phenotype A. The retina presents refined compensatory mechanisms and bystander effects in response to homeostatic and structural alterations (De Smet, 2017). Therefore, it can be hypothesized that in individuals classified as phenotype B, characterized by increased CRT, the occurrence of edema (Marques et al., 2020b) through disruption of the blood retinal barrier (BRB) may lead to a chronic retinal inflammatory environment (Madeira et al., 2015). On the other hand, individuals classified as phenotype C present progressive capillary closure (Marques et al., 2021b; Santos et al., 2021), leading to retinal ischemic events that have been shown to contribute to further neurodegenerative damage (Garcia-Martin et al., 2019).

Indeed, it has been proposed that since the early stages of diabetes, hyperglycemia induces retinal oxidative stress, low-grade inflammation, increased expression of glutamate, and changes in the expression of their receptors, leading to impaired neurotransmission and calcium homeostasis associated with degeneration of retinal ganglion cells (Santiago et al., 2006, 2009; Ishikawa, 2013). Likewise, this suggests that it is the retinal vasculature response to the early neurodegenerative changes that determines the course of retinopathy in each individual, whether predominantly characterized by alteration of the BRB and edema (in phenotype B) or by capillary closure and ischemia (in phenotype C).

The physiological, genetic, or other mechanisms that regulate this distinct interindividual response, which categorize each individual in its specific phenotype, remain to be elucidated. Importantly, previous work from our group has identified specific gene variants associated with the different risk NPDR phenotypes, which may explain underlying mechanisms that differentiate the three phenotypes (Simões et al., 2014). These data support our hypothesis, as phenotype B was shown to be associated with *ICAM1* gene variants, supporting the role of specific susceptibility to inflammation in this group of individuals. Likewise, phenotype C revealed an association with variants of the *PPARGC1A* gene, that are involved in endothelial damage, vascular leakage, and ischemia, and variants of the MTHFR gene, which contribute by inducing susceptibility to vascular damage (Simões et al., 2014).

Aschauer and colleagues have recently used OCT-angiography to demonstrate, in a 2-year longitudinal study, the relationship between subclinical changes in capillary perfusion and retinal neurodegeneration, showing that they may appear in parallel and in a progressive manner since the initial stages of NPDR (Aschauer et al., 2020). Likewise, taking this into account and the data here presented, additional longitudinal studies, taking advantage of OCT-angiography metrics, will clarify the correlation between retinal neurodegeneration and retinal capillary changes.

A limitation of this study is the focus on the initial stages of DR, allowing conclusions to be made only on the development of vision-threatening complications in people with T2D with ETDRS levels 20 and 35. Furthermore, the population studied is relatively well controlled, chosen using exclusion criteria such as excessive HbA_1C_ levels (>10%) and uncontrolled blood pressure. However, the use of these criteria guaranteed a relatively homogenous population. Another possible limitation is the relatively small number of people with T2D that completed the 5-year period of follow-up. However, the five-year duration of the study is of major value and offers new insights into the progression of retinal diabetic disease.

The observations here reported offer promising perspectives not only for personalized management of DR but also for development or identification of new therapeutic options that can also be used according to the phenotypic classification of each individual. Evidence that low-grade inflammation may play a key role as one of the responses to diabetic retinal neurodegeneration suggests that it can be envisaged as a target for the development of improved treatments (Madeira et al., 2015; Semeraro et al., 2019). This strategy could be used from the initial stages of DR, to control DR-associated neurodegeneration, limiting the microvascular-related inflammation that can itself lead to further retinal ganglion cell loss. Likewise, due to the central role of neurodegeneration in the course of DR, the use of neuroprotective agents can also be foreseen as a strategy for treating this disease (Simó et al., 2018) to delay the retinal microvascular response to neurogenerative events.

Finally, the findings here reported have a clear implication in the management of DR individuals, as they reinforce non-invasive methodologies that can be used to identify the eyes that are at risk of progression and developing vision–threatening complications, which are added value for improved management strategies of NPDR and allowing timely diagnosis of vision–threatening complications of diabetes. According to these observations and our previous reports (Marques et al., 2020b, 2021a,b), after diagnosis of NPDR and still in the initial stages of retinal disease, individuals classified with phenotype A showing a slower rate of retinal neurodegeneration are not expected to develop vision-threatening complications or DR severity progression and, therefore, do not require extensive follow-up at short intervals. On the other hand, T2D individuals classified as phenotype B or phenotype C should receive more attention, with relatively short follow-up intervals that facilitate a timely and efficient management of any sign of disease progression.

In summary, the classification of different retinopathy phenotypes in T2D is proposed as an easy to perform, reliable, and non-invasive method of individual categorization, facilitating a more accurate management and implementation of personalized medicine in DR.

## Data Availability Statement

The raw data supporting the conclusions of this article will be made available by the authors, without undue reservation.

## Ethics Statement

This study involving human participants was reviewed and approved by AIBILI’s Ethics Committee for Health (approval number CEC/007/16). The patients/participants provided their written informed consent to participate in this study.

## Author Contributions

MHM, IPM, SF, DT, ARS, and TS collected the data, analyzed, wrote, and reviewed and edited the manuscript. JF and CL assisted in the analysis and interpretation of the data. JC-V was the guarantor of this work and, as such, had full access to all the data in the study and takes responsibility for the integrity of the data and the accuracy of the data analysis. All authors have read and agreed to the published version of the manuscript.

## Conflict of Interest

JF reports being a member of Advisory Boards for Alimera, Allergan, Bayer, Bhoeringer, and No-vartis. JC-V reports being a consultant for Alimera Sciences, Allergan, Bayer, Gene Signal, Novartis, Pfizer, Precision Ocular Ltd., Roche, Sanofi-Aventis, Vifor Pharma, and Carl Zeiss Meditec. The remaining authors declare that the research was conducted in the absence of any commercial or financial relationships that could be construed as a potential conflict of interest.

## Publisher’s Note

All claims expressed in this article are solely those of the authors and do not necessarily represent those of their affiliated organizations, or those of the publisher, the editors and the reviewers. Any product that may be evaluated in this article, or claim that may be made by its manufacturer, is not guaranteed or endorsed by the publisher.

## References

[B1] Aschauer J. Pollreisz A. Karst S. Hülsmann M. Hajdu D. Datlinger F. (2020). Longitudinal analysis of microvascular perfusion and neurodegenerative changes in early type 2 diabetic retinal disease. *Br. J. Ophthalmol.* [Epub ahead of print]. 10.1136/bjophthalmol-2020-317322 33293271

[B2] Chhablani J. Sharma A. Goud A. Peguda H. K. Rao H. L. Begum V. U. (2015). Neurodegeneration in type 2 diabetes: evidence from spectral-domain optical coherence tomography. *Investig. Ophthalmol. Vis. Sci.* 11 6333–6338. 10.1167/iovs.15-17334 26436886

[B3] Cunha-Vaz J. Bernardes R. Santos T. Oliveira C. M. Lobo C. Pires I. (2012). Computer-aided detection of diabetic retinopathy progression. *Digital Teleret. Screen. Teleophthalmol. Pract.* 226 161–181. 10.1007/978-3-642-25810-7_6

[B4] Cunha-Vaz J. Ribeiro L. Lobo C. (2014). Phenotypes and biomarkers of diabetic retinopathy. *Prog. Retin. Eye Res.* 41 90–111. 10.1016/j.preteyeres.2014.03.003 24680929

[B5] De Smet M. D. (2017). Insights into the physiopathology of inflammatory macular edema. *Dev. Ophthalmol.* 58 168–177. 10.1159/000455279 28351051

[B6] Early Treatment Diabetic Retinopathy Study Research Group (2020). Grading DIABETIC retinopathy from stereoscopic color fundus photographs – an extension of the modified airlie house classification: ETDRS report number 10. *Ophthalmology* 127 S99–S119. 32200833 10.1016/j.ophtha.2020.01.030

[B7] Figueira J. Fletcher E. Massin P. Silva R. Bandello F. Midena E. (2018). Ranibizumab plus panretinal photocoagulation versus panretinal photocoagulation alone for high-risk Proliferative diabetic retinopathy (PROTEUS Study). *Ophthalmology* 125 691–700. 10.1016/j.ophtha.2017.12.008 29395119

[B8] Friedman S. M. Almukhtar T. H. Baker C. W. Glassman A. R. Elman M. J. Bressler N. M. (2015). Topical nepafenac in eyes with noncentral diabetic macular edema. *Retina* 35 944–956. 10.1097/IAE.0000000000000403 25602634 PMC4408212

[B9] Garcia-Martin E. Cipres M. Melchor I. Gil-Arribas L. Vilades E. Polo V. (2019). Neurodegeneration in patients with type 2 diabetes mellitus without diabetic retinopathy. *J. Ophthalmol.* 2019:1825819. 31485340 10.1155/2019/1825819 PMC6702840

[B10] Ishikawa M. (2013). Abnormalities in glutamate metabolism and excitotoxicity in the retinal diseases. *Scientifica (Cairo)* 2013:52894. 10.1155/2013/528940 24386591 PMC3872404

[B11] Khoshnood B. Mesbah M. Jeanbat V. Lafuma A. Berdeaux G. (2010). Transforming scales of measurement of visual acuity at the group level. *Ophthalmic Physiol. Opt.* 30 816–823. 10.1111/j.1475-1313.2010.00766.x 21205268

[B12] Kim K. Kim E. S. Kim D. G. Yu S. Y. (2019). Progressive retinal neurodegeneration and microvascular change in diabetic retinopathy: longitudinal study using OCT angiography. *Acta Diabetol* 56 1275–1282. 10.1007/s00592-019-01395-6 31401734

[B13] Klein R. Klein B. E. K. Moss S. E. (2001). How many steps of progression of diabetic retinopathy are meaningful? The Wisconsin epidemiologic study of diabetic retinopathy. *Arch. Ophthalmol.* 119 547–553. 10.1001/archopht.119.4.547 11296020

[B14] Lieth E. Gardner T. W. Barber A. J. Antonetti D. A. (2000). Retinal neurodegeneration: Early pathology in diabetes. *Clin. Exp. Ophthalmol.* 28 3–8. 10.1046/j.1442-9071.2000.00222.x 11345341

[B15] Lim H. B. Shin Y. I. Lee M. W. Koo H. Lee W. H. Kim J. Y. (2020). Ganglion cell – inner plexiform layer damage in diabetic patients: 3-year prospective longitudinal, observational study. *Sci. Rep.* 10:1470. 10.1038/s41598-020-58465-x 32001760 PMC6992712

[B16] Lobo C. Pires I. Alves D. Pappuru R. Ribeiro L. Cunha-Vaz J. (2018). Subclinical macular edema as a predictor of progression to central-involved macular edema in type 2 diabetes. *Ophthalmic Res.* 60 18–12. 10.1159/000486792 29510401

[B17] Madeira M. H. Boia R. Santos P. F. Ambrósio A. F. Santiago A. R. (2015). Contribution of microglia-mediated neuroinflammation to retinal degenerative diseases. *Mediat. Inflam.* 2015:673090. 10.1155/2015/673090 25873768 PMC4385698

[B18] Marques I. P. Alves D. Santos T. Mendes L. Lobo C. Santos A. R. (2020a). Characterization of disease progression in the initial stages of retinopathy in type 2 diabetes: A 2-year longitudinal study. *Investig. Opthalmol. Vis. Sci.* 61:20. 10.1167/iovs.61.3.20 32181799 PMC7401457

[B19] Marques I. P. Alves D. Santos T. Mendes L. Santos A. R. Lobo C. (2019). Multimodal imaging of the initial stages of diabetic retinopathy: different disease pathways in different patients. *Diabetes* 68 648L–653. 10.2337/db18-1077 30523027

[B20] Marques I. P. Kubach S. Santos T. Mendes L. Madeira M. H. de Sisternes L. (2021a). Optical coherence tomography angiography metrics monitor severity progression of diabetic retinopathy – 3-year longitudinal study. *J. Clin. Med.* 11:2296. 10.3390/jcm10112296 34070479 PMC8197493

[B21] Marques I. P. Madeira M. H. Messias A. L. Martinho A. C.-V. Santos T. Sousa D. C. (2021b). Different retinopathy phenotypes in type 2 diabetes predict retinopathy progression. *Acta Diabetol.* 58 197–205. 10.1007/s00592-020-01602-9 33025221 PMC7889686

[B22] Marques I. P. Madeira M. H. Messias A. L. Santos T. Martinho A. C. Figueira J. (2020b). Retinopathy phenotypes in type 2 diabetes with different risks for macular edema and proliferative retinopathy. *J. Clin. Med.* 9:1433. 10.3390/jcm9051433 32408522 PMC7290313

[B23] Meteyard L. Davies R. A. I. (2020). Best practice guidance for linear mixed-effects models in psychological science. *J. Mem. Lang.* 112:104092. 10.1016/j.jml.2020.104092

[B24] Narayan K. M. V. Boyle J. P. Geiss L. S. Saaddine J. B. Thompson T. J. (2006). Impact of recent increase in incidence on future diabetes burden: U.S., 2005-2050. *Diabetes Care* 29 2114–2116. 10.2337/dc06-1136 16936162

[B25] Nunes S. Ribeiro L. Lobo C. Cunha-Vaz J. (2013). Three different phenotypes of mild nonproliferative diabetic retinopathy with different risks for development of clinically significant macular edema. *Investig. Ophthalmol. Vis. Sci.* 10 4595–4604. 10.1167/iovs.13-11895 23745006

[B26] Oliveira C. M. Cristóvão L. M. Ribeiro M. L. Abreu J. R. F. (2011). Improved automated screening of diabetic retinopathy. *Ophthalmologica* 226:191. 21865671 10.1159/000330285

[B27] Ribeiro L. Pappuru R. Lobo C. Alves D. Cunha-Vaz J. (2018). Different phenotypes of mild nonproliferative diabetic retinopathy with different risks for development of macular edema (C-TRACER study). *Ophthalmic Res.* 59 59–67. 10.1159/000484666 29268280

[B28] Santiago A. R. Gaspar J. M. Baptista F. I. Cristóvão A. J. Santos P. F. Kamphuis W. (2009). Diabetes changes the levels of ionotropic glutamate receptors in the rat retina. *Mol. Vis.* 15 1620–1630. 19693289 PMC2728563

[B29] Santiago A. R. Rosa S. C. Santos P. F. Cristóvão A. J. Barber A. J. Ambrósio A. F. (2006). Elevated glucose changes the expression of ionotropic glutamate receptor subunits and impairs calcium homeostasis in retinal neural cells. *Investig. Ophthalmol. Vis. Sci.* 47 4130–4137. 10.1167/iovs.06-0085 16936133

[B30] Santos A. R. Mendes L. Madeira M. H. Marques I. P. Tavares D. Figueira J. (2021). Microaneurysm turnover in mild non-proliferative diabetic retinopathy is associated with progression and development of vision-threatening complications: a 5-year longitudinal study. *J. Clin. Med.* 10:214. 10.3390/jcm10102142 34063514 PMC8156148

[B31] Semeraro F. Morescalchi F. Cancarini A. Russo A. Rezzola S. Costagliola C. (2019). Diabetic retinopathy, a vascular and inflammatory disease: therapeutic implications. *Diab. Metabol.* 45 517–527. 10.1016/j.diabet.2019.04.002 31005756

[B32] Silk M. J. Harrison X. A. Hodgson D. J. (2020). Perils and pitfalls of mixed-effects regression models in biology. *PeerJ* 8:e9.

[B33] Simó R. Stitt A. W. Gardner T. W. (2018). Neurodegeneration in diabetic retinopathy: does it really matter? *Diabetologia* 61 1902–1912. 10.1007/s00125-018-4692-1 30030554 PMC6096638

[B34] Simões M. J. Lobo C. Egas C. Nunes S. Carmona S. Costa M. Â. (2014). Genetic variants in ICAM1, PPARGC1A and MTHFR are potentially associated with different phenotypes of diabetic retinopathy. *Ophthalmologica* 232 156–162. 10.1159/000365229 25324196

[B35] Soares M. Neves C. Marques I. P. Pires I. Schwartz C. Costa M. Â. (2017). Comparison of diabetic retinopathy classification using fluorescein angiography and optical coherence tomography angiography. *Br. J. Ophthalmol.* 101 62–68. 10.1136/bjophthalmol-2016-309424 27927677

[B36] Sohn E. H. van Dijk H. W. Jiao C. Kok P. H. Jeong W. Demirkaya N. (2016). Retinal neurodegeneration may precede microvascular changes characteristic of diabetic retinopathy in diabetes mellitus. *Proc. Natl. Acad. Sci. U.S.A.* 16:4113. 10.1073/pnas.1522014113 27114552 PMC4868487

[B37] Ting D. S. W. Cheung G. C. M. Wong T. Y. (2016). Diabetic retinopathy: global prevalence, major risk factors, screening practices and public health challenges: a review. *Clin. Exp. Ophthalmol.* 44 260–277. 10.1111/ceo.12696 26716602

[B38] Van De Kreekeid J. A. Darma S. Chan Pin Yin J. M. P. L. Tan H. S. Abramoff M. D. Twisk J. W. R. (2020). The spatial relation of diabetic retinal neurodegeneration with diabetic retinopathy. *PLoS One* 15:e023. 10.1371/journal.pone.0231552 32298369 PMC7161968

